# Prevalence and correlates of hazardous alcohol drinking and drug use among female sex workers and men who have sex with men in Mozambique

**DOI:** 10.1186/s12889-024-18273-8

**Published:** 2024-03-21

**Authors:** Cynthia Semá Baltazar, Rachid Muleia, Auria Ribeiro Banze, Makini Boothe

**Affiliations:** 1 Instituto Nacional de Saúde, Maputo, P.O. Box 264, Mozambique; 2 UNAIDS, Maputo, Mozambique

**Keywords:** Alcohol, Drugs, AUDIT-C, BBS, FSW, MSM, Mozambique

## Abstract

**Background:**

Hazardous drinking and drug consumption are associated with an increased risk of HIV due to the complex interplay of factors influencing decision-making capability, stigma and social marginalization. In this study, we explore the patterns of hazardous alcohol and drug use and correlates of risk factors among female sex workers (FSW) and men who as sex with men (MSM) in Mozambique.

**Methods:**

We conducted a secondary data analysis of bio-behavioral surveys (BBS) among FSW and MSM using a respondent-driven sampling methodology conducted in five main urban areas of Mozambique from 2019 to 20. The survey included a standardized questionnaire, where hazardous drinking was assessed (using AUDIT-C scores ≥ 4 for men, ≥ 3 for women) and drug use in the last year (FSW). Chi-squared test was used to analyze the association between socio-demographic and behavioral variables, and multivariate logistic regression measured the impact of the associated factors.

**Results:**

The prevalence of hazardous alcohol drinking was 47.1% (95% CI:44.8–49.5) for FSW and 46.5 (95% CI: 44.0–49.0) for MSM. Current drug use was reported in 13.3% of FSW. FSW engaging in hazardous alcohol drinking reported more sexual partners in the last month than those no reporting hazardous alcohol use (55.3% vs. 47,1%, *p* < 0.001), higher rates of self-reported STIs in the last year (62,5% vs. 48,2%, *p* < 0.001), physical (53.5% vs. 46.7%, *p* < 0.0001) and sexual violence (54.7% vs. 44.2%, *p* < 0.001), and HIV prevalence (55.2% vs. 44.2 *p* < 0.001). Among MSM with hazardous alcohol drinking, there was a higher prevalence of self-reported STIs (52.8% vs. 45.4%, *p* < 0.001), experiences of sexual violence (18.0% vs. 8.3%, *p* < 0.001), and HIV prevalence (53.0% vs. 46.3%, *p* < 0.001). In addition, FSW who reported illicit drug use were more likely to self-reported HIV own risk (14.2% vs. 9.7%), early start sexual activity (15.4% vs. 5.3%), self-reported STIs (17.9% vs. 10.2%), and experiences of both physical (17.4% vs. 7.0%) and sexual violence (18.6% vs. 8.9%).

**Conclusion:**

There is an immediate need for the introduction and integration of comprehensive substance use harm mitigation and mental health interventions into HIV prevention programs, particularly those targeting key populations in Mozambique.

## Background

HIV/AIDS is one of the world’s major public health challenges. Progress in the prevention and treatment of HIV/AIDS is not advancing at the same rate as the general population around the world, especially among the key populations with female sex workers (FSW) having a 30 times higher risk of acquiring HIV, and gay and other men who have sex with men (MSM), with 28 times greater risk than adults aged 15–49 years [1].

Some studies have documented that the elevated risk of acquiring HIV and other sexually transmitted infections (STI) is exacerbated by the use of illicit drugs and alcohol, particularly when multiple layers of stigma exist related to sex work, homosexuality, and violence as occurs in HIV key population [2–4]. Alcohol use can also co-occur with illicit drug use, and several studies reported a consistent and positive association of alcohol and drug use with sexual violence, and unprotected sexual intercourse among FSW and MSM [4, 5]. For these key populations, substance use often leads to poor decision-making and riskier behaviors, while also making them face more stigma and social exclusion, all of which contribute to the spread of HIV [6–8].

In sub-Saharan Africa, drinking is closely linked to unprotected sex, starting sex at a young age, and having multiple partners. Some research also points to higher risks when alcohol is used with other drugs [2]. The use of drugs and alcohol in FSW has been described as a coping mechanism to numb the challenges associated with the conditions of sex work and a facilitator for clients’ requests and wishes. Those data suggest that interventions to reduce drug and alcohol use may be important to prevent HIV and other sexually transmitted infections among high-risk. Thereby targeting these behavioral health factors, we can enhance the overall impact of public health initiatives designed to control and eliminate the spread of HIV [4, 6, 8, 9].

Furthermore, it is essential to consider the broader impact of substance use on mental health, social functioning, and overall well-being. The harmful use of substances not only increases the risk of HIV but also contributes to a range of mental health issues, disrupts social relationships, and deteriorates the overall quality of life of individuals. Addressing these broader implications is a critical component of public health initiatives aiming to control and eliminate the spread of HIV [10–13].

The WHO Global Information System on Alcohol and Health has developed The Alcohol Use Disorders Identification (AUDIT), an instrument to screen hazardous and harmful alcohol consumption, drinking behavior, and alcohol-related problems in healthcare settings and surveys [14]. The AUDIT tool can provide more accurate insights into the intricate links between alcohol consumption and risky sexual behavior [2].

Over the past few years, there has been increased advocacy to focus interventions among key populations, especially in low- and middle-income countries. The *Global AIDS Strategy 2021–2026: End Inequalities* emphasizes tackling inequalities, with equitable and equal access to HIV services to close the gaps preventing progress toward ending AIDS [15, 16]. Low engagement in services and subsequent low treatment outcomes among key population groups represent inequalities requiring specific attention.

Mozambique is one of the global countries most affected by the HIV epidemic, with an HIV prevalence of 12.5% in the general adult population, according to the recent Mozambique Population-based HIV Impact Assessments (PHIA) survey [17]. The 5th National Strategic Plan in Response to HIV/AIDS (2021–2025) prioritizes interventions for key populations, including FSW and MSM [18]. The contribution of alcohol and drug consumption to high-risk sexual behavior among FSWs and MSMs in Mozambique is not well documented.

In this context, recognizing the higher HIV prevalence among key populations, we conducted a secondary data analysis to describe patterns of substance use among FSW and MSM, who are particularly vulnerable to HIV infection and to assess the associated risk factors. This analysis was based on data from the second bio-behavioral surveys (BBS) conducted in Mozambique between 2019 and 2020. Understanding these patterns of hazardous alcohol and drug use is a crucial component in informing comprehensive HIV prevention efforts, especially in populations where the interplay of substance use and HIV risk is most pronounced.

## Methodology

### Survey design, participants, recruitment, and sampling

The second round of BBS among FSW and MSM in Mozambique was implemented between 2019 and 2020 in five major urban areas in the country (Maputo, Beira, Nampula, Zambezia, and Tete).

The two surveys used the same respondent driven sampling (RDS) methodology, a peer-to-peer sampling methodology widely used to recruit high-risk and hidden populations. In this method, initial respondent, identified as “seeds,” are recruited based on their role in the community, and they are issued coupons used to invite additional participants from within their network to participate in the study. RDS sampling methodology uses responses about a participant’s personal network size to apply adjustments to the sample data that represent the larger target population in the specific geographic location. The weights represent the probability of selection. An estimator is used to produce weights for each participant that are used to generate population proportions of estimates and corresponding standard errors. Details about the survey methodology are described elsewhere [19].

FSW were eligible to participate in the survey if they were 15 years of age or older (FSW aged 15–17 are officially considered sexually exploited minors and for the purpose of the study are classified as emancipated minors and were therefore allowed to provide written informed consent to participate in the survey); reported receiving money or goods in exchange for sex from someone other than a steady partner in the six months preceding the survey; live, work or socialize in the survey area; and had a valid referral coupon. Following the formative assessment, in each city we selected three seeds, and each seed was offered five coupons. In Maputo, Beira, Tete, Quelimane and Nampula, 419, 521, 519, 514 and 519 FWS were recruited to participate to participate in the study, respectively. The sample size for each city was calculated following WHO 2017 bio-behavioral survey guidelines for populations at higher risk of HIV infection [20]. The BBS surveys carried out with FSW in Mozambique included young women aged 15–17. This decision is well documented and was made after careful consideration, drawing on evidence from previous research indicating that this data is crucial for devising effective policies and programs targeted at this high-risk HIV group [19, 21–23].

MSM were eligible if they were biologically male; 18 years of age or older; reported oral or anal sex with another male in the 12 months preceding the survey; live, work or socialize in the survey area; and had a valid coupon. In both surveys Exclusion criteria included inability to provide informed consent or being under influence of alcohol and drugs. For MSM, the sample size calculation followed a similar approach as that for FWS. In the survey, 530 MSM were recruited in Maputo city, 527 in Beira, 559 in Tete, 525 in Quelimane, and 528 in Nampula. Further details on sample size calculation can be found in the report available at www.ins.gov.mz.

An interview using a standardized questionnaire was followed by two sequential rapid HIV tests (Determine™ and Uni-Gold™), along with pre-and post-test counseling on site, in line with Mozambique Ministry of Health guidelines [24].

#### Outcome variables

Bio-behavior variables were assessed using the WHO Biobehavioral Survey Guidelines for populations at risk for HIV [25]. Participants were asked about alcohol use and illicit drug consumption. Drug use was measured by asking participants the following questions, “During the last 12 months have you consumed any illicit drug (drugs without medical permission)?” and “Which drug did you use?” Responses included injectable (cocaine and heroin) and non-injectable drugs.

We adopted the Alcohol Use Disorders Identification (AUDIT) instrument developed by the WHO Global Information System on Alcohol and Health to screen hazardous and harmful alcohol consumption, drinking behavior and alcohol-related problems in healthcare settings and surveys [14]. While the AUDIT provides an in-depth screening for hazardous, harmful, and potentially dependent drinking with a scoring range of 0 to 40, the AUDIT-C offers a more streamlined assessment of severe binge drinking (heavy drinking and/or active alcohol abuse or dependence), suitable for rapid screening in primary care settings, with a scoring system ranging from 0 to 12. This distinction makes AUDIT-C particularly useful in our study for identifying individuals engaging in risky drinking behaviors [2, 14]. For the purpose of this study, The 3 AUDIT questions analyzed were “How often did you have a drink containing alcohol in the past year?”, “How many drinks containing alcohol did you have on a typical day when you were drinking in the past year? ”, and “How often did you have six or more drinks on one occasion in the past year?” a composite score was calculated depending on the responses.

We utilized a cut-off score of 4 for men and 3 for women, as recommended in clinical guidelines. This threshold was chosen to identify individuals with risky drinking behaviors effectively. For the purpose of our analysis, the AUDIT-C score was treated as a binary variable: scores at or above the cut-off indicated hazardous alcohol use, while scores below suggested lower-risk consumption. This binary categorization allowed us to clearly delineate between participants with potentially harmful drinking patterns and those without, facilitating a more focused investigation into the correlates of hazardous alcohol use within the study population.

#### Exploratory variables

Explanatory variables were chosen based on the literature and programmatic importance including socio-demographic variables, HIV infection, self-reported STIs in the past 12 months, experience with violence (physical and sexual), comprehensive HIV knowledge (which entails a thorough understanding of how HIV is transmitted, prevented, and managed), own HIV risk perception, and health-seeking behaviors: access of health and health services in the last 12 months, self-reported STI (symptoms or diagnosis), and HIV test. High-risk social and sexual behaviors including hazardous drinking (defined by-Audit C), drug use, age of first sex work experience, number of sexual partners during the last month and condom use were also included.

Self-reported STIs were assessed by responding “yes” to at least one of the following questions: “During the last six months, have you had an abnormal discharge from your vagina, anus or penis?”, “During the last six months, have you had a sore or ulcer near your vagina, anus or penis?,” and “In the last six months, did someone inform you that you had or could have a sexually transmitted infection?”

### Data analysis

Responses from the FSW and MSM, BBS surveys conducted during 2019–2020 were aggregated across survey-cities to produce pooled estimates for each population. Aggregate weighted estimates were computed using the RDS R package to analyze self-reported alcohol and drug use prevalence [26]. To produce aggregate weighted estimates, we considered the Gile’s successive sampling (RDS-SS) estimator, which accounts for the self-reported network sizes of participants, recruitment patterns and estimated size of the study population [27]. Details in the estimated size population for both FSW and MSM can be found in respective main reports. Unweighted bivariate analyses by Chi-square test were performed to identify risk factors associated with self-reported alcohol and drug use. The sample size of MSM reporting drug use was small (*n* = 36) and therefore were not included this sub-analysis.

All the analyses were done using the R statistical software 4.2.2 [28]. The code to run all the analyses is available upon request and approval.

### Ethical considerations

The bio-behavioral survey among key populations adhered to stringent ethical measures that were rigorously observed. Participants provided written informed consent, and the study protocols received approval from the Institutional Ethics Committee (CIE-INS) and Mozambican National Bioethics Committee for Health (CNBS). To ensure confidentiality, personal identifiers were not collected, and survey responses were anonymized. Data were stored securely, accessible only to authorized personnel, and were encrypted to maintain privacy. The research team received comprehensive training in ethical research practices, with an emphasis on safeguarding participant confidentiality given the sensitivity of the data. Moreover, the study was subject to regular ethical audits to ensure adherence to established ethical standards and protection of participants’ rights and welfare. Minor FSWs [14–16], were connected with the Office for Women and Children of the Mozambican police, under the Ministry of the Interior. This office facilitates access to the legal system within a secure environment for victims of violence. Additionally, these women were directed to the Mozambican League of Human Rights, which offers legal advice at no cost.

## Results

### Socio-demographic characteristics

A total of 2,567 FSW and 2,669 MSM from survey cities participated in the study. The majority of FSW were < 25 years old (60.5%), single/ (61.1%) and had secondary/high education (72.8%). Among MSM participants the majority were < 25 years (78%), single (89.2%) and had secondary or high education (99.0%). The overall HIV prevalence among FSW participants was 26.5% and among MSM was (7.0%). Main socio-demographic and health characteristics are summarized in Table 1.

**Table 1:** General characteristics and HIV status of FSW and MSM, 2019–2020

	FSW *N* = 2567	MSM *N* = 2669
**Age (years)**		
< 25	60.5	78.0
≥ 25	39.5	22.0
**Marital status**		
Single	61.1	89.2
Cohabitating/married	6.8	7.8
Widowed/divorced/Separated	32.1	2.9
**Education**		
No formal education/primary	27.2	1.0
Secondary/higher	72.8	99.0
**Place of residence**		
Maputo	17.7	18.9
Beira	19.0	16.5
Nampula	22.3	26.7
Tete	21.4	22.3
Quelimane	19.7	15.5
**HIV infection**		
Yes	26.5	7.0
No	73.5	93.0

### Hazardous alcohol and illicit drug among FSW and MSM

Among FSW in the five survey cities, nearly half (47,1%), reported hazardous drinking and 12.4% reported illicit drugs use. The prevalence of hazardous drinking among of MSM was 46.5% (Table 2).

**Table 2:** Prevalence of drug and alcohol use among FSW and MSM, 2019–2020

	FSW			MSM		
	n/N	%	RDS-weighted	n/N	%:	RDS- weighted
	Crude	Crude	(95% CI)	Crude	Crude	(95% CI)
**Consume alcohol in the past month**	1,420/2,565	55.3	53.2 (51.7–54.9)	1,559/2,664	58.5	57.2 (55.5–58.9)
**Hazardous drinking (AUDIT-C)**	1,255/2,567	48.9	47.1 (44.8–49.5)	1,304/2,669	48.9	46.5 (44.0–49.0)
**Illicit drug use last 12 months before the survey**	342/2,562	13.3	12.4 (9.0-15.8)	-	-	-

### Risk factors associated with hazardous drinking among FSW and MSM

Among the sub-sample of FSW who met AUDIT-C criteria for hazardous drinking, the median age was 24 years old (range:15–58). Hazardous drinking was more frequent in FSW participants from Maputo city (61.7% vs. 56.3% from Tete, 54.5% from Beira and 41.4% from Quelimane), > 25 years (54.3% vs. 42.5% for 15–24, *p* < 0.001) and those cohabitating or married (53.1% vs. 53.1% for widowed/divorced/separated and 43.5% for single, *p* < 0.001). More FSW participants with hazardous drinking behaviors reported having 2 or more clients in the last month (55.3% vs. 43.7% FSW with 1 client, *p* = 0.003), had higher STI self-reported (62,5% vs. 48,2%, *p* < 001) within the last month, had a higher own HIV risk perception (53.3% vs. 42.6% low), experience more physical (53.5% vs. 46.7%, *p* < 0.0001) and sexual violence (54.7% vs. 44.2%, *p* < 0.001) in the last 12 months preceding the survey, and were infected with HIV (55.2% vs. 44.2 *p* < 0.001) (Table 3).

MSM participants with hazardous drinking had a median age of 22 years old (range:18–61). Hazardous drinking was more frequent in MSM participants from Beira City (66.1% vs. 59.9% from Maputo, 59.9% from Tete, 26.3% from Nampula and 24.9 from Quelimane) younger MSM participants aged 19–24 years old (61.6% vs. 42.5% for 25 years and older, *p* < 0.001), and widowed/divorced/separated (65.9% vs. 59.3% cohabitating/married and 44.0% single). Most MSM participants with hazardous drinking had their first sexual experience with other men after 18 years of age (54.7% vs. 47.0 from 15 to 18, *p* < 0.001), had a higher HIV risk perception (53.4% vs. 45.8 low), higher self-reported STI (52.8% vs. 45.4%, *p* < 001. HIV infection was also more frequent among MSM participants reporting hazardous drinking (53.0% vs. 46.3 *p* < 0.001) (Table 3).

**Table 3:** Socio-demographic and behavior correlates of hazardous drinking (AUDIT-C) and risk factors among Female Sex Workers (FSW) and Men who have Sex with Men (MSM), Mozambique 2019–2020

	FSW (*N* = 1,255)				MSM (*N* = 1,304)			
	n/N Crude	% Crude	RDS-Unweighted (95%)	***p***-value	n/N Crude	% Crude	RDS-Unweighted (95%)	***p***-value
**City**				**< 0.001**				**< 0.001**
Maputo	305/492	62.0	61.7 (56.8–66.5)		323/530	6.9	59.9 (54.5–65.4)	
Beira	288/520	55.4	54.5 (48.9–60.1)		355/527	67.4	66.1 (61.3–70.8)	
Nampula	141/518	27.2	25.7 (21.5–29.8)		144/528	27.3	26.3 (21.7–30.8)	
Tete	294/521	56.4	56.3 (51.8–60.7)		340/559	6.8	59.8 (55.4–64.3)	
Quelimane	227/516	44.0	41.4 (36.1–46.6)		142/525	27.1	24.9 (20.5–29.3)	
**Median age**	24 (15–58)				22 (18–61)			
**Age group**				**< 0.001**				**< 0.001**
< 24 ^a^	647/1487	43.5	42.5 (39.5–45.4)		890/2041	43.6	42.5 (39.6–45.4)	
≥ 25	608/1080	56.3	54.3 (50.8–57.8)		414/628	65.9	60.6 (55.9–65.4)	
**Marital status**				**< 0.001**				**< 0.001**
Single	683/1522	44.9	43.5 (40.6–46.5)		1045/2269	46.1	44.0 (41.3–46.7)	
Cohabitating/married	100/183	54.6	53.1 (44.8–61.3)		131/202	64.9	59.3 (51.2–67.3)	
Widowed/divorced/ separated	471/859	54.8	52.8 (48.9–56.7)		62/90	68.9	65.9 (55.9–75.8)	
**Education**				0.015				0.114
No formal education/primary	357/668	53.4	51.4 (47.0-55.7)		7/17	41.2	33.8 (10.6–56.9)	
Secondary/higher	856/1,786	47.9	46.3 (43.5–49.1)		651/1,538	42.3	41.4 (38.3–44.6)	
**Work aside from sex work**				0.006	-	-	-	
Yes	403/759	53.1	52.8 (48.6–56.9)		-	-	-	
No	850/1,803	47.1	44.9 (42.2–47.6)		-	-	-	
**Perception of HIV risk**				**< 0.001**				**< 0.001**
Low	245/575	42.6	42.7 (38.1–47.3)		536/1,116	48.0	45.8 (42.2–49.4)	
High	537/1,007	53.3	51.4 (47.8–54.9)		529/968	54.7	53.4 (49.5–57.3)	
**Previous HIV testing**				**< 0.001**				**< 0.001**
Yes, in the past 12 months	361/647	55.8	53.8 (49.5–58.2)		293/583	5.3	47.0 (42.2–51.7)	
Ever	509/1,041	48.9	47.5 (43.9–51.1)		646/1,291	5.0	48.2 (44.7–51.6)	
Never	123/377	32.6	30.5 (25.3–35.7)		163/459	35.5	34.7 (29.5–39.8)	
**Comprehensive HIV knowledge**				0.065				0.955
Yes	583/1,241	47.0	43.8 (40.6–47.1)		58/118	49.2	46.9 (36.8–56.9)	
No	599/1,181	50.7	50.4 (47.2–53.6)		1237/2,519	49.1	46.8 (44.2–49.4)	
**Age at first sexual experience** ^**b**^				0.873				**< 0.001**
Less than 15 years	429/866	49.5	47.6 (43.7–51.5)		95/186	51.1	49.8 (41.4–58.1)	
15–17	621/1,283	48.4	47.0 (43.7–50.2)		257/518	49.6	47.9 (42.7–53.0)	
> 18	196/400	49.0	47.0 (41.4–52.5)		175/305	57.4	54.7 (48.1–61.4)	
**No. of sexual clients during the last month** ^**c**^				**0.003**				**< 0.001**
0	339/692	49.0	47.1 (42.7–51.5)		34/109	31.2	31 (21.5–40.5)	
1	601/1,298	46.3	43.7 (40.4–46.8)		81/247	32.8	34.2 (26.8–41.6)	
> 2	315/575	54.8	55.3 (50.7–59.8)		1,155/2,222	52.0	49.3 (46.5–52.0)	
**Condom use at last sexual encounter**				0.638	-	-	-	
Yes	339/692	49.0	47.1 (42.7–51.5)		-	-	-	
No	601/1298	46.3	43.7 (40.5–46.9)		-	-	-	
**Self-report STI in the last 12 months**				**< 0.001**				**< 0.001**
Yes	435/776	56.1	54.7 (50.7–58.7)		245/437	56.1	52.8 (47.4–58.2)	
No	818/1,782	45.9	44.2 (41.4–46.9)		1,057/2,223	47.6	45.4 (42.6–48.2)	
**Sought health care in the past 12 months**				0.010				
Yes	717/1,399	51.3	50.6 (47.4–53.7)		25/1,219	51.3	48.3 (44.7–51.8)	
No	538/1,165	46.2	43.6 (40.3–46.9)		678/1,444	47.0	45.1 (41.8–48.3)	
**HIV infection**				**< 0.001**				**< 0.001**
Yes	386/677	57.0	55.2 (50.9–59.5)		129/218	48.0	53.0 (44.9–61.0)	
No	806/1762	45.7	44.2 (41.4–47.0)		1,174/2,446	59.2	46.3 (43.4–48.6)	
**Experienced physical violence, last 12 months**				**< 0.001**				0.074
Yes	736//1,341	54.9	53.5 (50.4–56.5)		92/186	49.5	49.2 (40.8–57.6)	
No	519/1,224	42.4	40.3 (37.1–43.6)		1,212/2,479	48.9	46.3 (43.7–48.9)	
**Experienced sexual violence in the last 12 months**				**< 0.001**				0.075
Yes	545/975	55.9	54.3 (50.6–57.8)		23/43	53.5	47.8 (31.9–63.6)	
No	710/1,590	44.7	43.2 (40.2–46.1)		1,280/2,620	48.9	46.5 (43.9–49.1)	

^*a*^ FSW included SEA 15–24 years old and MSM includes 18–24 years old

^*b*^ For MSM, age of first sexual encounter with another man

^*c*^ Analysis only includes FSW ad MSM and who reported that are sex workers

### Risk factors associated with illicit drug use among FSW

Among the sub-sample of FSW participant who use illicit drug use in the last 12 months before the survey the median age was 22 years old (range 15–22). Illicit drug use in the past year was more frequent among FSW from Maputo City (25.5% vs. 13.7% from Beira, 10.5% Tete, 8.4% Quelimane and 6.3% from Nampula *p* < 0.001), and among FSW in a union (15.5% vs. 13.0% single and 10.7% widowed/divorced/separated, *p* = 0.003). More FSW that used illicit drugs had a high perception of their own HIV risk (14.2% vs. 9.7% low perception, *p* = 0.005) and have their first sexual experience when younger than 15 years old (15.4% vs. 12.6% for 15–17 years old and 5.3% for more than 24 years old, *p* < 0.001). More FSW who used drugs self-reported an STI in the last year (17.9% vs. 10.2% those did not, *p* < 0.001), and experienced physical (17.4% vs. 7.0% those that did not, *p* < 0.001) and sexual violence (18.6% vs. 8.9% those that did not, *p* < 0.001) in the last year before the survey (Table 4).

**Table 4:** Socio-demographic and behavioral correlates of drug use among Female Sex Workers (FSW) participants, Mozambique 2019–2020

	FSW who use drug, past 12 months			
	n/N:		%:	***p***-value
	Crude	Crude	Weighted (95% CI)	
**City**				**< 0.001**
Maputo	129/492	26.2	25.5 (20.9–30.6)	
Beira	76/520	14.6	13.7 (10.2–17.2)	
Nampula	33/518	6.3	6.3 (3.9–8.7)	
Tete	55/519	9.5	10.5 (0.2–20.8)	
Quelimane	49/516	1.6	8.4 (5.7–11.1)	
**Median age**	**22 (15–55)**			
**Age group**				< 0.296
15–24	207/1,486	13.9	13.1 (11.5–14.8)	
≥ 25	135/1,079	12.5	11.4 (9.4–13.4)	
**Marital status**				**0.003**
Single	207/1,523	13.6	13.0 (11.0–15.0)	
Cohabitating/married	38/183	20.8	15.5 (9.8–21.2)	
Widowed/divorced/separated	97/861	11.3	10.7 (8.4–12.9)	
**Education**				0.586
No formal education/primary	86/668	12.9	12.8 (7.4–13.4)	
Secondary or higher	245/1,789	13.4	12.3 (10.4–14.6)	
**Work aside from sex work**				0.866
Yes	100/759	13.2	12.2 (9.2–15.1)	
No	242/1,806	13.4	11.8 (9.7–13.8)	
**Perception of HIV risk**				**0.005**
Low	62/575	1.8	9.7 (6.8–12.5)	
High	160/1,009	15.9	14.2 (11.5–16.7)	
**Previous HIV testing**				**0.004**
Yes, in the past 12 months	75/648	11.6	10.6 (7.9–13.3)	
Ever	164/1,042	15.8	14.5 (11.1–16.3)	
Never	37/378	9.8	9.3 (6.0-12.6)	
**Comprehensive HIV knowledge**				0.427
Yes	155/1,242	12.5	11.9 (9.8–14.1)	
No	161/1,186	13.6	12.3 (10.2–14.5)	
**Age at first sexual experience**				**< 0.001**
Less than 15 years	145/869	16.7	15.4 (12.6–18.1)	
15–17	162/1,283	12.6	11.8 (9.8–13.8)	
> 18	34/400	8.5	5.3 (5.3–11.5)	
**No of sexual clients during the last month**				0.024
0	111/694	15.9	16.0 (12.9–19.3)	
1	152/1,298	11.7	10.5 (8.4–12.4)	
> 2	79/576	13.7	12.7 (9.5–15.8)	
**Condom use at last sexual encounter**				0.049
Yes	261/2,056	12,7	11.6 (9.9–13.2)	
No	81/506	16.0	15.9 (12.3–19.4)	
**Self-report STI in the last 12 months**				**< 0.001**
Yes	146/776	18.8	17.9 (14.6–21.2)	
No	193/1,782	1.8	10.2 (8.5–11.8)	
**Sought health care in the past 12 months**				0.073
Yes	202/1,399	14.4	13.7 (11.5–15.8)	
No	140/1,165	12.0	11.1 (9.1–13.2)	
**HIV infection**				0.843
Yes	93/676	13.8	11.9 (9.3–14.6)	
No	237/1,762	13.5	12.8 (10.9–14.6)	
**Experienced physical violence, last 12 months**				**< 0.001**
Yes	251/1,341	18.7	17.4 (14.9–19.7)	
No	91/1,224	7.4	7.0 (5.37–8.7)	
**Experienced sexual violence in the last 12 months**				< **0.001**
Yes	194/975	19.9	18.6 (15.7–21.4)	
No	148/1,590	9.3	8.9 (7.2–10.7)	

## Discussion

Our findings indicate a high prevalence of hazardous alcohol consumption among FSW and MSM where close to half are considered at risk according to the AUDIT-C scale, which is consistent with the literature [5, 6, 29, 30]. For FSW, hazardous drinking use may serve as a coping mechanism to deal with the nature of their work (stress, violence, criminalization and stigmatization), while for MSM, alcohol consumption may serve as a mechanism to deal with stigma and related stress [4, 30]. These results emphasize the need for alcohol risk reduction programmes for key populations specifically focused on the adoption of safer drinking practices integrated into HIV prevention packages.

In our study, hazardous drinking was more frequent among MSM with younger age. This likely mirrors social behavior related to alcohol use in the general population to enhance pleasure, to be more social, and/or life histories of traumatic experiences such as sexual orientation-based discrimination and childhood sexual abuse [31]. Several studies demonstrate alcohol use in younger ages to be associated with a greater number of lifetime sexual partners, non-protected sexual intercourse, elevated levels of depressive symptoms, and alcohol abuse later in life [29, 31, 32]. Within younger MSM, where there is an increased risk of acquiring HIV compared to their older counterparts, alcohol use preceding sexual intercourse represents an increase risk for the acquisition of HIV and other STI [7, 31, 33].

Nearly half (47.1%) of our FSW participants reported hazardous drinking, a figure that aligns closely with the 41% prevalence of hazardous/harmful/dependent alcohol use among FSWs reported in 32 low- and middle-income countries. This rate is substantially higher than the global prevalence of alcohol use disorders among women in the general population, which stands at 5.1% [5]. Unweighted pooled estimates conducted in our study demonstrate that FSW with hazardous alcohol consumption have an increased number of sexual partners, a higher occurrence of self-reported STI, higher perception of their own HIV risk, and have higher HIV prevalence. Other studies demonstrate that hazardous alcohol consumption likely account for the positive association between frequency of drinking and increased number of clients [2]. Previous studies have demonstrated long-term links between alcohol consumption and HIV infection in the general population [4, 15, 34]. Particularly among FSW Alcohol affects decision-making about negotiating for safer sex, which can increase risk of HIV and other STI transmission [8]. In addition hazardous heavy drinking and drug use have also been associated with poor adherence to antiretroviral therapy (ART) among HIV positive people and the interaction between all those substances usually leads to a higher susceptibility to co-morbidities and opportunistic infections [8, 35]. The high burden of alcohol use among FSWs also carries serious health and social implications, as excess alcohol consumption is linked with a range of adverse physical and mental health outcomes, underscoring the need for targeted interventions in this population [4, 8, 36].

Physical and sexual violence is recognized as a widespread public health problem and a violation of human rights. We found FSWs that use alcohol are enmeshed in a dynamic of physical and sexual violence victimization. A prior study conducted among FSW in the country found a high prevalence of physical and sexual violence, confirming the need for specific interventions to address this vulnerability [23]. Furthermore, it’s important to note that while sex work is not criminalized in Mozambique [37], it remains highly stigmatized, and the legal system does not include specific protections for sex workers, creating additional challenges for those affected by alcohol-related issues.

“While our study found a prevalence of illicit drug use among FSW participants at 13.3%, it is noteworthy that the systematic review by Iversen et al. identified a higher pooled prevalence of lifetime illicit drug use among sex workers globally at 35% [38]. It is important to note that the prevalence of drug use among FSWs is a complex issue that is influenced by various factors such as social, economic, and cultural determinants. Consequently, the prevalence of drug use among FSWs may vary across different regions and countries. However, the risk profile of FSWs who use illicit drugs in our study is consistent with what has been observed in other studies among key populations (KP) in Mozambique. These studies consistently show that drug use tends to occur more frequently in younger individuals with a higher number of sexual partners, and it is associated with higher HIV and STI prevalence [19, 39]. Several additional studies demonstrate that there is a strong relationship between substance use and unsafe sexual practices that increases the risk for HIV and STIs [7, 30, 36, 40–42]. Additionally, FSW may use substances as a coping mechanism to numb the challenges associated with their sexual practices, which can also contribute to hazardous drinking [36, 40–42].

Our study had some potential limitations. First, we measured the alcohol and drug use consumption by self-report thus, the data might be under-reported due to social desirability bias. Second, due to missing data we were not able to assess the drug consumption among MSM. Third, like other cross-sectional surveys, we could not assess cause-and-effect relationships, for example it was not possible to assess whether hazardous drinking behaviors and illicit drug use preceded sexual risk behaviors. Fourth, the FSW and MSM network assessed in the study may be missing important sub-groups (E.g. FSW and MSM with higher economic status) and therefore may not capture the specific drug and alcohol behaviors of these sub-populations. In addition, the analysis of pooled results from across the survey cities may affect the representativeness of sample to the general KP population in the survey cities. Finally, these findings need to be interpreted with caution and cannot be generalized to the entire MSM and FSW population in the survey cities nor at the national level. Although we were able to find significant association between the outcome variables and the exposure, we did not provide the strength of the association, considering that the analysis was more descriptive than inferential. Therefore, results should be interpreted with caution. Additionally, it should be noted that RDS is only valid within each area.

## Conclusion

The data from this study indicate that hazardous drinking use are associated with behaviors that increase the risk of HIV among FSW and MSM, and illicit drug among FSW. As these two groups are among the most vulnerable populations for HIV, there is a need to integrate substance abuse screening, referral, and treatment into HIV prevention and treatment services. Additional studies are needed to further explore the relationship between alcohol consumption, illicit drug use and mental health issues among these populations to inform efforts that address the inequalities of the HIV epidemic.

## Data Availability

The Mozambique National Institute of Health (INS) provides full access to all study information and data sets in their data repository. This resource is available to researchers who fulfill the criteria for accessing confidential data. The data originates from the Bio-Behavioral Survey (BBS) studies. Researchers interested in these data sets can contact the authors or obtain further information through the INS website at www.ins.gov.mz.
